# Vesicular Trafficking Defects, Developmental Abnormalities, and Alterations in the Cellular Death Process Occur in Cell Lines that Over-Express *Dictyostelium* GTPase, Rab2, and Rab2 Mutants

**DOI:** 10.3390/biology3030514

**Published:** 2014-08-25

**Authors:** Katherine Maringer, Entsar Saheb, John Bush

**Affiliations:** Department of Biology, University of Arkansas at Little Rock, 2801 S. University Ave., Little Rock, AR 72205, USA; E-Mails: ejsaheb@ualr.edu (E.S.); Jmbush@ualr.edu (J.B.)

**Keywords:** *Dictyostelium*, GTPase, Rab2, vesicular trafficking, cell death, development

## Abstract

Small molecular weight GTPase Rab2 has been shown to be a resident of pre-Golgi intermediates and required for protein transport from the ER to the Golgi complex, however, the function of Rab2 in *Dictyostelium* has yet to be fully characterized*.* Using cell lines that over-express DdRab2, as well as cell lines over-expressing constitutively active (CA), and dominant negative (DN) forms of the GTPase, we report a functional role in vesicular transport specifically phagocytosis, and endocytosis. Furthermore, Rab2 like other GTPases cycles between an active GTP-bound and an inactive GDP-bound state. We found that this GTP/GDP cycle for DdRab2 is crucial for normal *Dictyostelium* development and cell–cell adhesion. Similar to Rab5 and Rab7 in *C. elegans*, we found that DdRab2 plays a role in programmed cell death, possibly in the phagocytic removal of apoptotic corpses.

## 1. Introduction

The Rab family of small GTPases plays an important role in the regulation of vesicle trafficking between intracellular organelles [1]. Rabs cycle between an active GTP-bound state and an inactive GDP-bound state, and associate with transport vesicles through C-terminal lipid modifications [2,3]. After vesicle docking at target membranes, GTP hydrolysis, via the intrinsic GTPase activity of the Rab, converts the Rab to its GDP-bound form [3]. Rabs play an important role in endocytic and exocytic membrane trafficking and have been implicated in the control of both fusion and fission events that are required for the proper maturation of endosomes as well as phagocytic compartments [4,5,6]. For instance, Rab4, Rab5, and Rab11 associate with early endosomes [6,7], Rab7 and Rab9 associate with late endosomes [8,9,10]. At least four members of the Rab family associate with the secretory pathway including Rab1, Rab3, Rab6, and Rab11 [7,11,12,13]. This Rab mediated control of vesicular trafficking seems to be conserved across many species including *Dictyostelium discoideum* [5].

There are 54 Rab related GTPases that have been identified in *Dictyostelium*. Of them, few have been characterized. Rab7 regulates phagosome-lysosome membrane traffic [14], Rab11 has been implicated in the internalization of particles [7], and RabD has been shown to regulate macropinocytosis and endo-lysosomal fusion [15]. Several Rabs have also been implimented in development including Rab8 [3,16,17,18,19], Rab13 [3,20,21,22], Rab10 [3,23], and Rab3A [3,24].

*Dictyostelium* offers numerous advantages as an experimental model system in which to study development. It possesses a simple and well-defined life cycle consisting of a vegetative phase and a developmental phase (reviewed by Loomis 1975 [25]). *Dictyostelium* is an amoeba for most of its life, upon starvation a very interesting developmental program is induced during which individual cells stream together by chemotaxis to form a multicellular tissue [26]. During a morphogenetic process involving cell migration and cellular morphogenesis a simple mound is transformed into a slug or pseudoplasmodium which establishes a simple developmental pattern. The slug then develops into a fruiting body which consists of multiple cell types to include spores and stalk cells called a sporocarp [27]. The stalk cells are a result of cell death [28]. *Dictyostelium* cell death is similar to classical apoptosis in that some cytoplasmic and chromatin condensation occurs but differs from apoptosis because it involves massive vacuolization and, interestingly, lacks DNA fragmentation [28,29].

In this study we explore the role of *Dictyostelium* GTPase Rab2 which is 81% identical to human Rab2. To explore the role of Rab2 in *Dictyostelium*, we analyzed cells overexpressing DdRab2, as well as cells expressing constitutively active (CA), and dominant negative (DN) forms of the GTPase. We demonstrate that DdRab2: (1) has an effect on the rate of endocytosis; (2) plays a role in multi-cellular development; and (3) may play a role in induced cell death.

## 2. Experimental

### 2.1. Cells and Culture Conditions

For all experiments, *D. discoideum*, wild-type strain AX4, pDneo2a-GFP, Rab2-GFP, Rab2(CA)-GFP, and Rab2(DN)-GFP expressing cell lines were grown axenically at 21 °C in shaking culture at 150 rpm in HL5 medium:1% oxoid proteose peptone, 1% glucose, 0.5% yeast extract (Fisher Biotech, Fair Lawn, NJ, USA), 2.4 mM Na_2_HPO_4_, and 8.8 mM KH_2_PO_4_, pH 6.5. This media was supplemented with 300 mg/mL of streptomycin sulfate and 100 mg/mL of ampicillin (Sigma, St. Louis, MO, USA). Additionally, for the transfected cells, HL5 medium was supplemented with 10 mg/mL of G418 (Invitrogen, Carlsbad, CA, USA). To minimize background fluorescence from the HL5 medium, cells were incubated in “Loflo” medium for 24 h before the experiments [30].

### 2.2. Creation of GFP Tagged Rab2 Cell Lines

*Dictyostelium* Rab2 cDNA was subjected to PCR using primers that had a *SalI* restriction enzyme to the sense primer, and an *XhoI* restriction enzyme to the anti-sense primer. The resulting PCR products were ligated into the TA vector (Invitrogen) and sequenced for both errors and the presence of the *SalI* and *XhoI* sites. The Rab2 PCR TA product was then digested with *SalI* and *XhoI*, purified and ligated into the pDneo2a-GFP vector (N-terminus GFP tag) previously cut with *SalI* and *XhoI*. The resulting construct (Rab2-GFP) was sequenced for errors and reading frame confirmation. The constructs were then electrotransfected into *Dictyostelium* AX4 cells and selected with G418 antibiotic (Invitrogen).

Dominant negative (DN), and constitutively active (CA) forms of the Rab2 protein were created by changing a key amino acid asparagine (N) to isoleucine (I) at amino acid position 118 (dominant negative) and by changing amino acid glutamine (Q) to leucine (L) at amino acid 64 (constitutively active) using the Stratagene QuickChange^®^ II Site-Directed Mutagenesis Kit (Stratagene, La Jolla, CA, USA). The resulting mutant constructs were then sequenced to confirm the single change in the Rab2 protein.

### 2.3. Western Blotting

For western blotting to verify expression of all constructs at comparable levels, 5 × 10^6^ cells were harvested, re-suspended in 1 mL of double distilled water, and transferred to a 1.5 mL tube. 150 µL of fresh lysis buffer with 0.3 gm of glass beads was added and centrifuged for 10 min at 2200 rpm. 50 µL of the supernatant was transferred to a fresh 1.5 mL tube and 50 µL of fresh 2 X SDS-loading dye was added. The sample was heat treated for 10 min at 99 °C, subjected to 10% SDS-PAGE, and transferred to PVDF membrane (Millipore Cor. Bedford, Cat. no IPVH00010) using a Hoefer Transfer Unit as described by Bush *et al.* 1994 [31]. Blots were incubated with primary antibodies (1:2000 dilution of a mouse monoclonal anti-GFP antibody) in antibody buffer (20 mM Tris, pH 7.5, 140 mM NaCl, 0.05% Tween 20, 1% powdered milk). Samples were then washed; incubated with goat anti-mouse secondary antibody conjugated to horse radish peroxidase (Phototope^®^-HRP Western Blot Detection Kit, New England Biolabs, Ipswich, MA, USA); and visualized by exposing the membrane to X-ray film for 60 s and the film was developed using standard developing methods.

### 2.4. Phagocytosis, Pinocytosis, Exocytosis, and Recycling Assays

Phagocytosis was measured using fluorescent rhodamine isothiocynate latex beads (RITC-latex beads, Sigma Aldrich). Fluid phase pinocytosis, exocytosis, and recycling rates were measured using rhodamine isothiocynate-dextran (RITC-dextran, Sigma Aldrich) as described by Rivero and Maniak, 2006 [32]. Data was run through a one-way ANOVA to test for significance at *p* > 0.05. Cells were viewed and photographed using the BrightLine^®^TXRED Filter Set on a Nikon 2000SE microscope with IPLab 3.7 software (Scanalytics, Inc., Fairfax, VA, USA) with 1000 times magnification.

### 2.5. Lysosomal Visualization: LysoTracker Staining

LysoTracker^®^ (Molecular Probes) is a dye used in *D. discoideum* to mark acidic organelles, mainly lysosomes [33]. Cells were harvested and allowed to adhere on cover slips in HL5 medium. The medium was then replaced with fresh medium containing 100 nM LysoTracker^®^ and incubated for 30 min at room temperature. Fluorescence was visualized using the BrightLine^®^ DAPI filter set, where lysosomes fluoresced blue, and the BrightLine^®^GFP filter set for comparison with GFP localization.

### 2.6. Endosome Visualization: RITC-Dextran Loading

Rhodamineisothiocynate-dextran (RITC-dextran, Sigma Aldrich) is a fluid internalized in endosomes but not degraded [34]. In *Dictyostelium*, the red fluorescence of RITC was used to mark endosomes. First, cells were harvested and allowed to settle in a 35 × 10 mm Petri dish containing 2 mL of HL5 medium. Next, 40 µL of 100 mg/mL RITC-dextran was added to the cells and incubated for 60 min. Cells were then collected, washed, and allowed to settle on a glass cover slip. All cell lines were photographed using the BrightLine^®^ TXRED filter set to visualize endosomes, and the BrightLine^®^ GFP filter set to visualize GFP fluorescence.

### 2.7. Development Assay

For developmental studies, cells were grow axenically in HL5 medium on a rotary shaker (160 rpm) to 1 × 10^9^ cells/mL and harvested by centrifugation. After repeated washing with developing buffer (5 mM Na_2_HPO_4_, 5 mM Na_2_HPO_4_, 5 mM KH_2_PO_4_, 1 mM CaCl_2_, 2 mM MgCl_2_), cells were re-suspended in the same buffer at a density of 2 × 10^8^ cells/mL. Then 200 mL of the cell suspension was spread evenly on a 100 mm KK2 agar plate using a sterile glass spreader. The plates were wrapped with plastic wrap including a wet paper towel, inverted and incubated at 22 °C for the desired time to monitor the different developmental stages. This method was adapted from Fey *et al.* 2007 [30]. Photographs of multicellular development were taken using a model AM 4201, 25× microscope (Lutron Instruments, Tamil Nadu, India).

### 2.8. Cell Cohesion Assay

Cell cohesion assays were performed as described by Wong *et al.* 2002, Secko *et al.* 2006, and Desbarats *et al.* 2004 [35,36,37]. To determine developmental cell cohesion, vegetative cells were centrifuged at 700 g for 4 min, washed in KK2, re-suspended in KK2 at 2 × 10^7^ cells/mL and starved in rotary-agitated suspension (175 rpm) at 22 °C for 4 h. The cell suspension was diluted to a density of approximately 2.5 × 10^6^ cells/mL and aggregates were dispersed by vigorously vortexing for 15 s. Aggregates were allowed to reform while rotating on a platform shaker at 180 rpm at room temperature. At the indicated times, the number of non-aggregated cells, including singlets and doublets, were scored using a haemocytometer and the number of aggregating cells was determined by subtracting this number from the total number of cells and was expressed as a percentage of the total.

To assess the EDTA-resistant cell–cell adhesion, cells were harvested and re-suspended in KK2 and starved in a rotary shaker at 22 °C for 4 h. Cells were then collected and re-suspended in KK2 + 10 mM EDTA to inhibit EDTA-sensitive cohesion. Aggregates were dissociated by brief vortexing, and samples (0.2 mL) were placed in plastic tubes and rotated vertically at 180 rpm at room temperature for cell re-association. The percentage of cell aggregation was monitored at regular intervals for 60 min. Experiments were generally repeated three or more times.

### 2.9. Flow Cytometry Assay

To induce cell death, vegetative cells in late exponential growth phase were harvested and washed twice with SB (soerensen phosphate buffer: 14.5 mM KH_2_PO_4_, 2.5 mM Na_2_HPO_4_, pH 6). Approximately 5 × 10^5^ cell/ mL were suspended in 1 mL of SB containing 3 mM cAMP (Sigma) in a flask and incubated for 8 h at 22 °C. Cells were washed with 1 mL of SB which was replaced afterward with either 1 mL of SB or 1 mL of SB containing 0.1 mM differentiation inducing factor DIF (1-(3-Chloro-2,6-dihydroxy-4-methoxyphenyl)-1-hexanone, Sigma) and incubated for 30 h at 22 °C. The cells were re-suspended with a titer of 1× 10^6^ cell/ mL in SB in 300 µL aliquots. Test tubes for 1 µg/mL Propidium Iodide (PI) staining were used and incubated for 10 min at 22 °C with no wash. Cytometry analysis was then performed on a FACSCalibur cytometer from Becton Dickinson using CellQuest software [31].

### 2.10. Determining Cell Viability Due to ATP Levels

The number of viable cells in culture was determined based on measuring the quantity of ATP, which is a signal of the presence of active cells [37]. The CellTiter-Glo^®^ Luminescent Cell Viability Assay kit (Promega) was used for this test. Approximately 5 × 10^5^ vegetative cells were harvested and re-suspended in 100 µL of 10 mM MES (Sigma). Next, the cells were transferred into opaque-walled 96-well plates [38]. Cells were then lysed by adding 100 µL of the CellTiter-Glo reagent and mixing on a shaker for 2 min. Microtiter^®^ Plate Illuminometer B36580 with Revelation MLX software measured the plates after incubation at room temperature for 10 min for ATP levels present in the cellular extracts [39].

### 2.11. Cellular Re-Growth Assay

Vegetative cells in the late exponential growth phase were harvested and washed twice with SB. Approximately 5 × 10^5^ cells/mL were added in 1 mL of SB containing 3 mM cAMP in a two Lab-Tek chamber and incubated for 8 h at 22 °C. The cells were then washed with 1 mL of SB that was later replaced with either 1 mL of SB or 1 mL of SB containing 0.1 mM DIF and incubated for 30 h at 22 °C. From each chamber, 0.5 mL of SB was removed and replaced with 1 mL of HL-5 medium and incubated for 72 h at 22 °C. Cells were detached and counted using hemocytometer and phase contrast optics. Finally, the ratio of the number of cells re-growing in the DIF chamber to the number of cells in the control chamber was calculated [32].

### 2.12. Cell Growth Rate

All cell lines were grown in HL5 medium until a density of 1–3 × 10^4^ cells/ mL was obtained. Cellular growth was determined by counting the cells every 24 h over five days using a hemacytometer (Hausser Scientific, Horshman, PA, USA).

## 3. Results and Discussion

### 3.1. Results

#### 3.1.1. DdRab2 Plays a Role in the Regulation of Vesicular Trafficking

**Figure 1:**
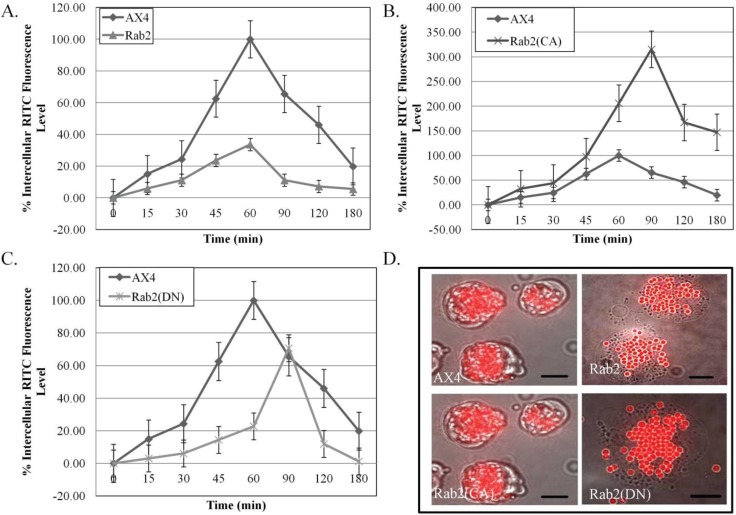
Phagocytosis is altered in DdRab2 over-expressing and DdRab2(CA) cell lines. (**A**–**C**) Graphical representation with standard error of the internalization of latex beads to quantify phagocytosis over 180 min showing significant differences (*p* > 0.05) in internal fluorescence in the Rab2 (▲) and Rab2(CA) (×) cell lines over the AX4 (♦) parent strain. Data is presented as the average of three individual experiments with relative fluorescence to AX4 which is being considered 100% at 60 min. (**A**) The Rab2 over-expressing cell line showed a significant decrease in internal fluorescence representing phagocytosis compared to the WT-AX4 cell line; (**B**) The Rab2(CA) expressing cell line showed a significant increase in internal fluorescence representing phagocytosis compared to WT-AX4 cells. Rab2(CA) expressing cells interestingly showed a decrease in the rate of phagocytosis compared to WT-AX4 cells, not reaching their maximum internalization until the 90 min time point compared to the WT-AX4 cells reaching maximum internalization at 60 min; (**C**) Rab2(DN) expressing cell lines showed no significant difference in the internal fluorescence representing phagocytosis as compared to AX4 cells, however, the rate of phagocytosis in this cell line was decreased reaching maximum phagocytosis at 90 min compared to the WT-AX4 cells reaching maximum phagocytosis at 60 min; (**D**) Overlaid images of bright field with visualization of red latex beads in AX4, Rab2 over-expressing, Rab2(CA), and Rab2(DN) cell lines. The image shows a decrease in the amount of beads compared to the AX4 in the Rab2 over-expressing cell line, and an increase in the amount of beads in the Rab2(CA) expressing cell line confirming the amount of internal fluorescence representing phagocytosis observed in the graphical representation. Scale Bar: 5 µm.

*Dictyostelium discoideum* has proven to be a useful system in which to investigate the molecular mechanisms regulating endosomal and lysosomal membrane trafficking. Rab GTPases have been implemented in playing an important role in the regulation in vesicle trafficking between intracellular organelles [10]. The following experiment was performed in order to determine the role of DdRab2 in the endocytic pathway. For all experiments, cell lines were constructed to over-express DdRab2, DdRab2(CA), and DdRab2 (DN). Western blot analysis confirmed that all constructs were expressed at comparable levels (data not shown). The rate of phagocytosis was measured by assaying DdRab2 over-expressing, DdRab2(CA), and DdRab2(DN) cell lines for their ability to internalize latex beads compared to the parent strain AX4 cell line. Graphical representation shown in Figure 1A shows a decrease in the internalization of latex beads in the DdRab2 over-expressing cell line. At the 60 min time point when the parent strain AX4 cells have a phagocytosis rate of 100%, the DdRab2 over-expressing cell line has a phagocytosis rate of only 30%, a 70% decrease. Conversely, in the DdRab2(CA) cell line (Figure 1B) the rate of phagocytosis was increased. At the 60 min time point, the rate of phagocytosis shows an increase of 100% in the DdRab2(CA) cell line. The parent strain AX4 cells reach 100% phagocytosis at 60 min while the DdRab2(CA) cell line reach their maximum rate of phagocytosis after 90 min. Similar to the DdRab2 over-expressing cell line, the DdRab2(DN) cell line (Figure 1C) shows a decrease in the rate of phagocytosis. Up to the 60-min time point, DdRab2(DN) cells show as much of an 80% decrease compared to the AX4 cells. While the AX4 cells reach 100% phagocytosis after 60 min, the DdRab2(DN) cell line does not reach its maximum rate of phagocytosis until 90 min, similar to that of the DdRab2(CA) cell line. Figure 1D shows a photographic representation of the graphical results. The increase in the internalization of latex beads in the DdRab2(CA) cell line, and decrease in the internalization of latex beads in the DdRab2 over-expressing and DdRab2(DN) cell lines is apparent confirming the graphical evidence.

**Figure 2:**
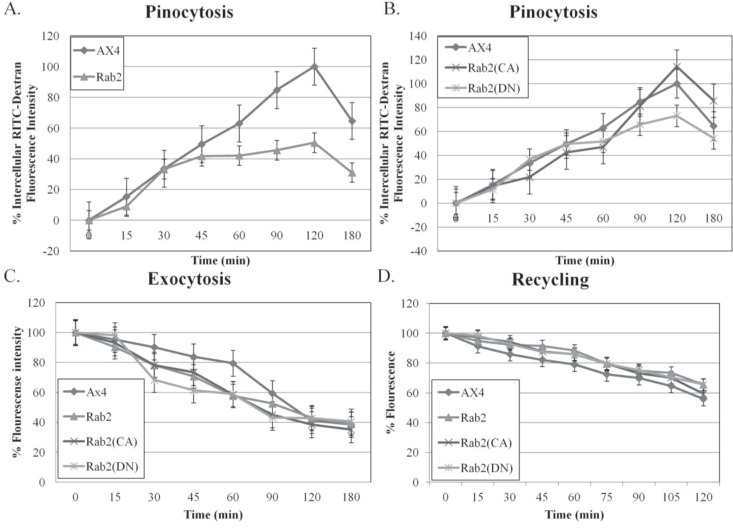
Pinocytosis and Exocytosis are altered in DdRab2 over-expressing, DdRab2(CA), and DdRab2(DN) cells. (**A**,**B**) Graphical representation with standard error of the rate of pinocytosis in Rab2 over-expressing, Rab2(CA), and Rab2 (DN) cell lines compared to the parent strain AX4 cells. Data for pinocytosis, exocytosis, and recycling is the average of three independent experiments with a *p*-value > 0.05. Cell lines were incubated in HL5 medium containing RITC-dextran (100 mg/mL) over 180 min. Data are presented as relative fluorescence to AX4 which is considered 100% RITC-dextran internalization at 120 min. At the indicated times, cells were washed by centrifugation and accumulated RITC-dextran was measured using a fluorimeter. (**A**) The intercellular fluorescence representing pinocytosis in the Rab2 over-expressing cell line is decreased compared to AX4 cells, however, this decrease was not significantly significant; (**B**) The intercellular fluorescence representing pinocytosis in the Rab2(CA), and Rab2(DN) cell lines. The Rab2(CA) cell line shows a slight increase in pinocytosis at the 120-min time point while the Rab2(DN) cell line shows a slight decrease in the rate of pinocytosis after 120 min compared to the AX4 cells (increase and decrease not statistically significant); (**C**) Graphical representation with standard error of the rate of exocytosis. After 180 minof RITC-dextran accumulation, cells were washed and re-suspended in growth medium. At the indicated times, the remaining intracellular RITC- dextran was measured. Rab2, Rab2(CA), and Rab2(DN) cells did not show a significant change in intercellular fluorescence representing exocytosis; (**D**) Graphical representation with standard error of RITC-Dextran recycling over 120 min showed no significant difference in recycling amongst all cell lines.

Given the dramatic effect of wild type and mutant DdRab2 overexpression on phagocytosis, further study into the role of DdRab2 in the endocytic pathway was done. The ability of the DdRab2 over-expressing, and DdRab2 mutant cell lines to internalize the fluid phase marker rhodamine isothiocyanate-dextran (RITC-dextran) was measured as a way to study pinocytosis. Analysis of the data shown in Figure 2A shows a decrease of 55% in the internalization of RITC-dextran by the wild type DdRab2 overexpressing cell line as compared to the parent strain AX4 cells. Interestingly, the DdRab2(CA) cell line shows a slight increase of approximately 20% while the DdRab2(DN) cell line showed a slight decrease of approximately 35% in the internalization of RTIC-dextran compared to the AX4 cells (Figure 2B).

The rates of exocytosis and fluid phase recycling of RITC-dextran were also quantified. The ability of the DdRab2 over-expressing, and DdRab2 mutant cell lines to release the marker was slightly decreased compared to the AX4 cell line; however this difference was at its greatest with just a 20% decrease. The rate of fluid phase recycling of RITC-dextran showed no difference in any of the cell lines compared to the AX4 parent strain (Figure 2C,D).

**Figure 3:**
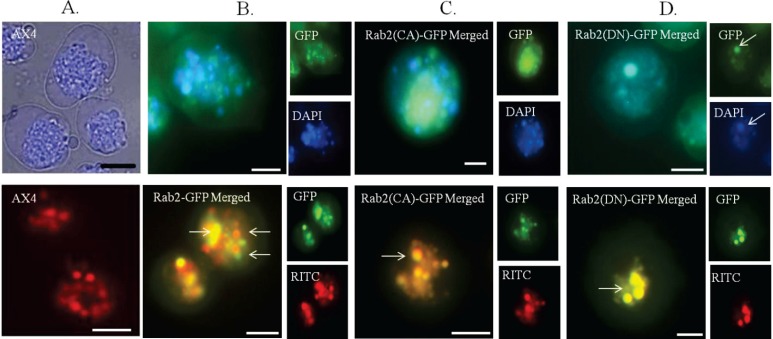
DdRab2(DN) associates with lysosomes and endosomes. (**A**–**D** top panel) Distribution of LysoTracker^®^ dye in the AX4 cell line (**A**) and overlaid images of LysoTracker^®^ blue (DAPI) with GFP visualization in the Rab2-GFP (**B**) over-expressing, Rab2(CA)-GFP (**C**), Rab2(DN)-GFP (**D**) cell lines. The Rab2-GFP over-expressing (**B**), and Rab2(CA)-GFP (**C**) cell lines do not show association with the lysosomes. Rab2(DN)-GFP cells show co-localization with the lysosomes. (**A**–**D** bottom panel) The vesicles of the endocytic system from endosome to lysosome are shown as red staining membranes due to the uptake of RITC-dextran after 60 min of treatment. (**A**) WT-AX4 cells with the endosomes shown in red. (**B**) Rab2-GFP over-expressing, and Rab2(CA)-GFP (**C**), cells show partial association with the endosomal system (indicated by arrows). The Rab2(DN)-GFP (**D**) cells show complete association with the endosomal system. These images support a role for Rab2 in vesicular trafficking. Scale Bar: 5 µm.

Next, a microscopic approach was taken to investigate the role of DdRab2 in vesicular trafficking. Using GFP tagged DdRab2, DdRab2(CA), and DdRab2(DN) proteins, cells were observed and subjected to fluorescence microscopy to visualized and determine possible association of DdRab2 with lysosomes and endosomes. Cells expressing DdRab2-GFP, DdRab2(CA)-GFP, and DdRab2(DN)-GFP were stained with LysoTracker (Molecular Probes) which stains acidic organelles including lysosomes [33] or cells were allowed to internalize endosomal marker RITC-dextran followed by photography to observe the overlap of the GFP tagged proteins with the lysosomes or endosomes. Figure 3 (top) shows cells stained with LysoTracker blue, Figure 3 (bottom) shows cells after the internalization of RITC-dextran. DdRab2-GFP over-expressing, and DdRab2(CA)-GFP cells do not appear to co-localize with lysosomes (Figure 3B,C top) however, they do appear to partially co-localize with endosomes (Figure 3B,C bottom). Interestingly, DdRab2(DN)-GFP cells (Figure 3D) appear to co-localize with both the lysosomes and the endosomes. This microscopic and graphical evidence suggest that DdRab2 is involved in the regulation of vesicular trafficking.

#### 3.1.2. Rab2 Mutants Display Defects in Development and Adhesion

Since analysis of data previously described suggest that DdRab2 plays a role in the regulation of endocytosis, a look into the effect of DdRab2 on development was next examined. This investigation was initiated because several GTPases including Rac1A [40], Rac1B [41], Rac1C [40], and RasGEFB [42] that were defective in endocytosis also displayed defects in development. Furthermore, Rab2 has been identified as an essential protein for intracellular proliferation of *B. abortus* [43,44]. In mice, neurons treated with Rab2 show enhanced adhesion followed by a dramatic increase in neurite growth suggesting that Rab2 plays an important role in neuronal differentiation [45].

**Figure 4:**
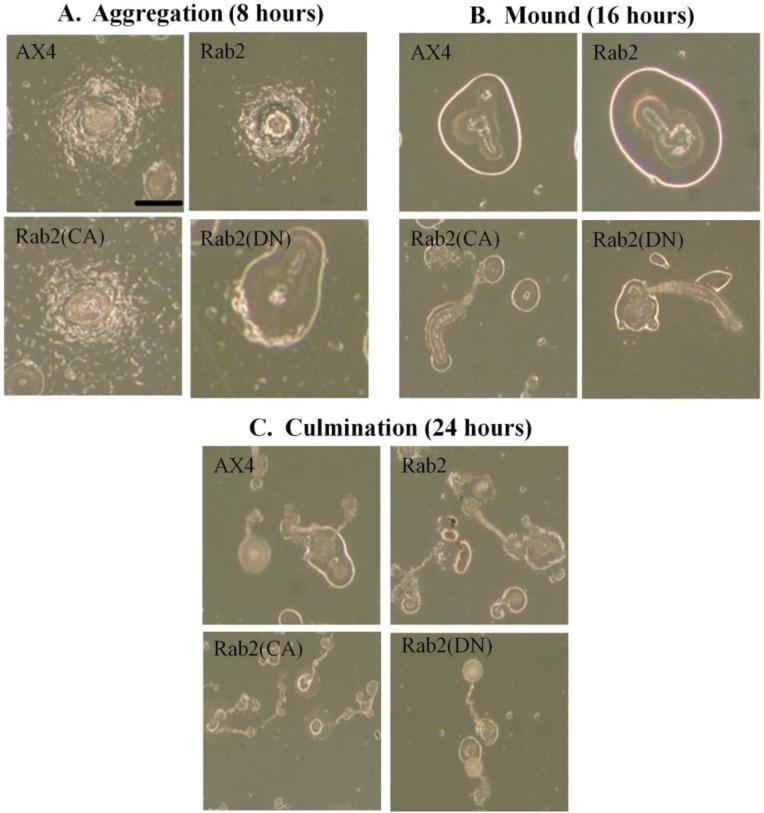
Over-expressing mutant forms of DdRab2 has an effect on the developmental process. (**A**) Representative photomicrographs of aggregation after 8 h of incubation at 22 °C on KK2 agar plates. Loose aggregates are visible in the AX4, Rab2, and Rab2(CA) cell lines after 8 h while the Rab2(DN) cells had already aggregated and formed a mound after 8 h; (**B**) Representative photomicrographs of mound formation after 16 h. After 16 h, wild type AX4, and Rab2 over-expressing cells show normal mound formation. Rab2(CA) cells have formed elongated mound structures. Rab2(DN) cells appear to have already formed fruiting bodies after 16 h; (**C**) Representative photomicrographs of fruiting body formation after 24 h. WT-AX4 and Rab2 over-expressing cell lines developed into fruiting bodies in the absence of nutrition after 24 h. Rab2(CA) and Rab2(DN) cell lines had formed fruiting bodies after 16 h and were still present after 24 h. The Rab2(CA) cell line appears to have formed a larger number of fruiting bodies that were smaller in size. Scale Bar: 1 mm.

The initial experiments focused on the phases of development that were observed following incubation at 22 °C on KK2 agar plates after 8 h (aggregation), 16 h (mound formation), and 24 h (culmination). Control cells expressing GFP alone were identical to the AX4 cells in both timing and morphology of cell development (data not shown) as were DdRab2 over-expressing cells (Figure 4A–C). In contrast to these data, DdRab2(CA) expressing mutants formed aggregates that were slightly larger than the control and seemed to attract a greater number of cells into each aggregate mound (Figure 4A). These aggregates then appeared to dissociate into smaller groups giving rise to deformed mound structures that were very elongated and smaller in size compared to AX4 cells (Figure 4B). After 24 h, the DdRab2(CA) cell line had formed small fruiting body structures as well as a greater number of fruiting bodies correlating to the increase in mound formation (Figure 4C).

On the other hand, DdRab2(DN) mutants also appear to be defective in its developmental program. After 8 h, DdRab2(DN) mutants had already completely aggregation, forming mound structures (Figure 4A). After 16 h, these mutants had formed fruiting body structures (Figure 4B). After 24 h, the fruiting bodies were still present in the DdRab2(DN) mutants although, the spore containing head appeared larger than that of the AX4 cells (Figure 4C).

**Figure 5:**
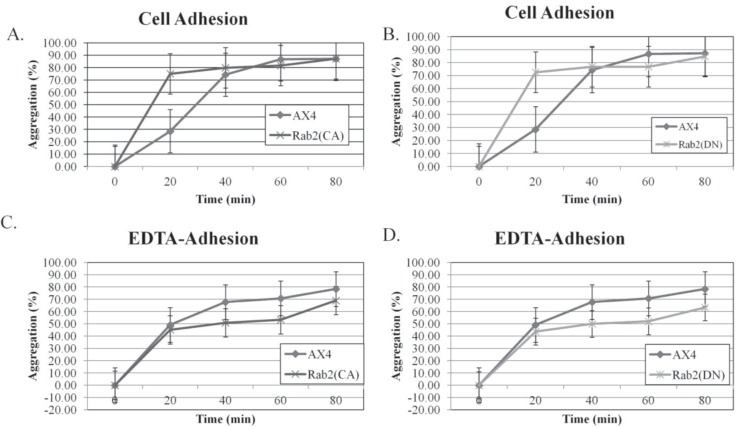
Mutant forms of DdRab2 show an initial increase in cell–cell adhesion. AX4, pDneo2a-GFP, Rab2, Rab2(CA), and Rab2(DN) cells were washed free of antibiotics and starved for 4 h in KK2 in a shaking suspension. Cells were then dissociated by vortexing, and cell re-association was monitored over time with or without the addition of 10 mM EDTA. Cells were not in aggregates at time zero and the percentage of cell re-association was calculated by scoring non-aggregated cells with a haemocytometer [percentage aggregation = (total number of cells − non-aggregating cells)/total number of cells]. Data represent the mean of three independent experiments *±* standard error with a *p*-value > 0.05. (**A**,**B**) cell adhesion of developing cells of the Rab2(CA) (**A**), and Rab2(DN) (**B**) cell lines in KK2 buffer. Both the Rab2(CA) and Rab2(DN) cell lines show an initial increase (not significant) in cell adhesion at the 20-min time point, after 20 min, cell adhesion is similar to that of the controls. (**C**,**D**) Cell adhesion of developing cells in the presence of 10 mM EDTA. Rab2(CA) (**C**), and Rab2(DN) (**D**) cell lines show a slight decrease (not significant) in cell–cell adhesion in the presence of EDTA.

Defects in development particularly the aggregation stage caused by Rabs or Rab-like proteins have been previously linked to alterations with cell–cell adhesion [3,37,46]. Rab5, Rab7, and Rab11 have been implicated in the coordinated regulation of cell–cell adhesion [47]. We wanted to determine if the defects in development observed could be due to increased cell–cell adhesion. Adhesivety was measured as described previously by Wong, 2002, and Secko *et al.* 2006 [35,36]. Cells were washed free of antibiotics and starved in KK2 buffer for 4 h. Cells were dissociated by vigorous vortexing to break up existing aggregates and then allowed to re-associate on a rotary shaker [35,36]. Some re-association was detected in the AX4 cells after 20 min (approximately 30%) (Figure 5A,B). The re-association of the DdRab2(CA), and DdRab2(DN) cells was much greater after 20 min at approximately 75% re-association. However, by 40 min, DdRab2(CA), DdRab2(DN), and AX4 cells were roughly equal in re-association (Figure 5A,B). It has been previously stated that early in the aggregation stage of development, *Dictyostelium* cells acquire EDTA-resistant cell binding sites [37]. To assess the EDTA-resistant cell–cell adhesion, cells were incubated in KK2 buffer + 10 mM EDTA following the four hour KK2 buffer incubation to inhibit EDTA-sensitive cohesion, and aggregates were counted as described above. Both the DdRab2(CA), and DdRab2(DN) cells show a decrease in EDTA-sensitive adhesion after 20 min compared to the AX4 cells (Figure 5C,D). Cellular response to cAMP was also examined to explain the abnormal development observed in the DdRab2(CA), and DdRab2(DN) cells however, no detectable difference was seen in these cell lines compared to the AX4 cells (data not shown). This led us to believe that the initial increase in cell–cell adhesion observed plays a role in the increased rate of development in the DdRab2(DN) cell line, and the abnormal development in the DdRab2(CA) cell line.

#### 3.1.3. Cell Death Is Decreased in Rab2 Mutants

The abnormalities observed in the development program in DdRab2 over-expressing and mutant cell lines (CA, and DN mutations) led us to investigate the role if any of the DdRab2 protein in *Dictyostelium* cell death. To investigate this hypothesis, all cell lines were starved, subjected to DIF treatment, PI staining and cell death quantification using flow-cytometry.

**Figure 6:**
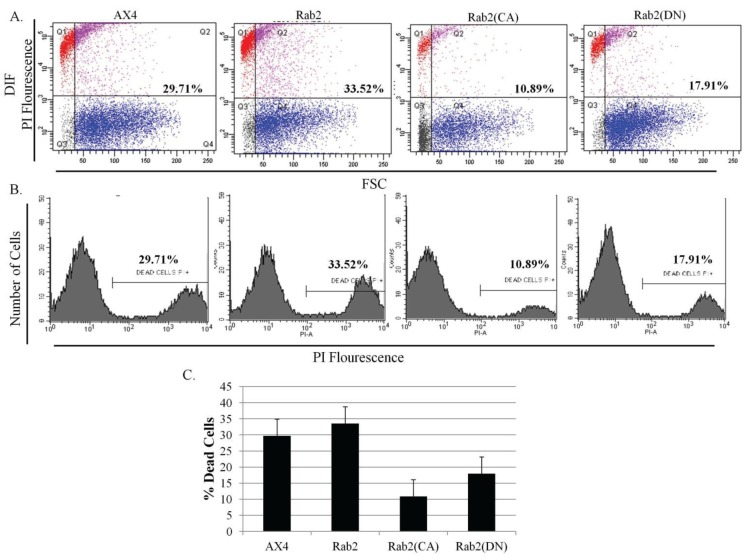
Cell death levels are reduced in cells over-expressing mutant forms of DdRab2. (**A**–**C**) Cell death quantification using flow cytometry analysis. Data are the result of three independent experiments with a *p*-value > 0.05 (**A**) WT-Ax4, Rab2 over-expressing, Rab2(CA), and Rab2(DN) cells were subjected to starvation and cAMP, and incubated with or without DIF for 6 h. Cells were then stained with 1 µg/mL Propidium Iodide for 10 min. Fluorescent PI-positive cells were quantified using flow cytometry. Dot plot data with side scatter and forward scatter showed dead cells distinct from living cells. Rab2(CA) cell line shows a significant decrease, and Rab2(DN) cell lines show a slight decrease (not significantly different) in the levels of cellular apoptosis as compared to the WT-AX4 cells. (**B**) Histogram of the dot plot data; (**C**) Quantification for DIF treatment cells undergoing apoptosis. Rab2(CA) showed a significant decrease, and Rab2(DN) cell lines showed a slight decrease (not significant) in the rate of apoptosis as compared to the WT-AX4 cell line.

**Figure 7:**
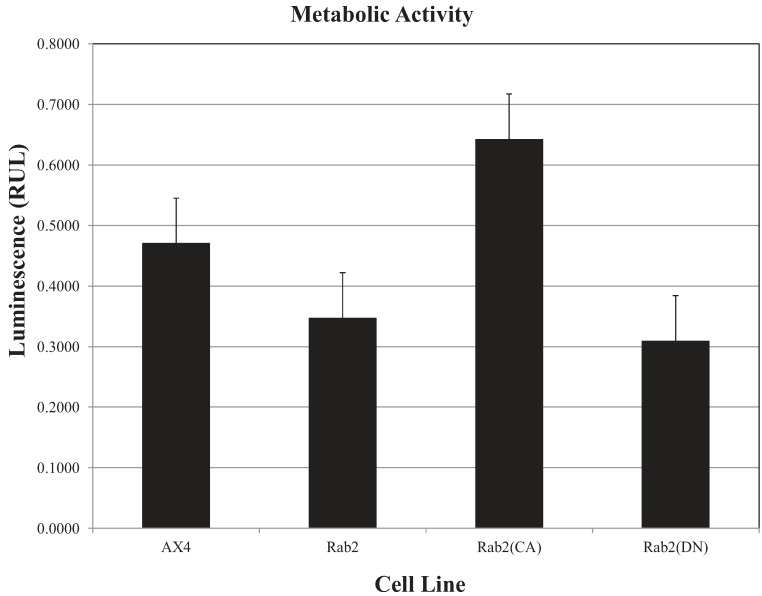
ATP levels are affected in DdRab2 over-expressing, and mutant cell lines. To measure the ATP levels, luminescence was recorded 10 min after CellTiter-Glo^®^ reagent was added to cells in a 96-well plate. Data represent the average of three independent experiments with a *p*-value > 0.05. There was a decrease (not significant) in the luminescent signal from the Rab2 over-expressing, and Rab2(DN) cells, and an increase in Rab2(CA) cells as compared to the AX4 cell lines.

Analysis of the results in Figure 6A–C show the percentage of induced cellular death in the parent AX4 cells and DdRab2 over-expressing cells are roughly equivalent. Interestingly, the DdRab2(CA) mutant in cells appears to protect those cells from cell death. The measured cell death in those cells was 20% less than that of the AX4. The mutation of the DdRab2(DN) cells appeared to protect those mutant cells from death only 12% less than the wild typeAX4 cells. Furthermore, the viability of the cells was determined by measuring the amount of ATP, an indicator of cellular metabolic activity [38]. Figure 7 shows that the DdRab2 over-expressing cell line has a slight decrease in metabolic activity correlating with its cell death levels. DdRab2(CA) show an increase in metabolic activity correlating to the decrease in cell death shown in Figure 6. Interestingly, in DdRab2(DN) cells we observed a decrease in metabolic activity and a decrease in cellular apoptosis indicating that although cell death has been decreased, these cells are not as metabolically active and are not healthy cells.

#### 3.1.4. Rab2 Has an Increased Growth Rate and Ability to Re-Grow after Induced Differentiation

The functional role of DdRab2 in protecting cells from cellular death as determined by the previous data analysis using the mutants leads to further investigations into the role of DdRab2 on re-growth of cells after treatment with DIF-1. DIF-1 is a putative morphogen that induces stalk cell formation in *Dictyostelium* [48]. Cells were treated as described by Rivero and Maniak, 2006 [32].

**Figure 8:**
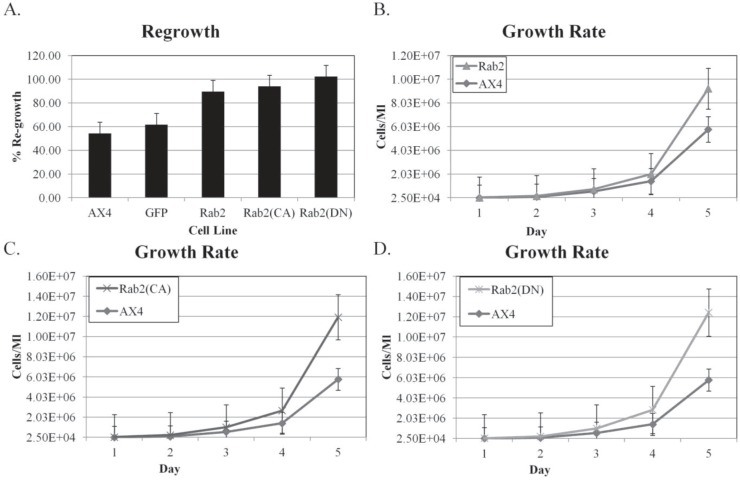
DdRab2 over-expressing, DdRab2(CA), and DdRab2(DN) cells have increased re-growth and growth rates compared to controls. (**A**) Percentage of re-growth of the mutant cells after 30 h of treatment with DIF followed by 72 h incubation with HL5. The ratio of the number of cells re-growing in the DIF chamber to the number of cells in the control chamber was calculated. Data are the average of three indepent experiments with a *p*-value > 0.05. Rab2 over-expressing, Rab2(CA), and Rab2(DN) cell lines had a significantly increased level of re-growth compared to the AX4 and pDneo2a-GFP cell lines. (**B**–**D**) Growth rate of Rab2 over-expressing, Rab2(CA), and Rab2(DN) cell lines. Cells were incubated in HL5 medium. At the same time each day cells were counted. Data are the average of three independent experiments with a *p*-value > 0.05. All cell lines have a significant increase in growth rate compared to the AX4 and pDneo2a-GFP (not shown). The increase in growth rate correlates to the increase in re-growth.

First, the ratio of the number of re-growing cells compared to the control was calculated. As shown in Figure 8A, DdRab2, DdRab2(CA), and DdRab2(DN) expressing cells have an increased re-growth rate than the control AX4 cells. Interestingly, the DdRab2 over-expressing cells have an increased re-growth rate however, these cells were not shown to protect from cell death and they had a decrease in metabolic activity so the increase in re-growth could be due to an increased growth rate. The DdRab2(CA), and DdRab2(DN) cell lines increase in re-growth was to be expected given their decrease in cellular apoptosis and increase in metabolic activity.

To test the idea that the increase in the re-growth observed was due in part to an increased growth rate of the cells, the growth rate was then calculated. Cells were suspended at a density of 2 × 10^4^ and counted each day at the same time for 5 days and then the growth rate was graphed (Figure 8B–D). All cell lines had an increased growth rate as compared to the AX4 cells. The increase in growth rate correlates to the increase in re-growth shown in Figure 8A. Interestingly, the DdRab2(DN) cell line has the fastest growth rate at almost 10X that of the AX4 cells and their ability to re-grow after treatment with DIF-1 reached above 100% indicating that they not only completely re-grew after treatment but they had begun to proliferate.

### 3.2. Discussion

#### 3.2.1. Alterations in Endocytosis

The Rab family of small GTPase proteins plays an important role in trafficking of vesicles and in determining specificity of vesicle fusion within the cell [4,49,50]. Human Rab2 has been implicated in Golgi-ER retrograde transport [51,52], but the mechanism by which Rab2 controls this transport is unknown. In addition, it is not clear whether Rab2 is involved in other aspects of endocytosis or vesicle trafficking. In this study we show that *Dictyostelium* Rab2 plays a role in vesicular trafficking, specifically, CA forms of DdRab2 increase the rate of phagocytosis, and pinocytosis while the opposite effect is seen in DN forms of the protein. We also show through microscopy of the GFP tagged proteins that DdRab2 partially associates with endosomes and lysosomes. Interestingly, the DN version of the DdRab2 protein shows complete association with the endosomes and lysosomes. This may be due to the GDP-bound Rab being unable to leave the membranes. Our findings implicating DdRab2 in vesicular trafficking are similar to the study by Lu, *et al.* 2008 [52] in which they suggest that *C. elegans* UNC-108 (the mamalian Rab2 homolog) is envolved in endocytosis, specifically endosomal trafficking and lysosome maturation [52]. Another study also performed in *C. elegans* by Chun, *et al.* 2008 [50], demonstrates that Rab2 is envolved in postendocytic trafficking [50]. Rab2 has also been implicated in phagosome maturation in both *C. elegans*, and mammalian cells [44,53]. While our results show that DdRab2 is involved in the regulation of the rate of pinocytosis and phagocytosis, and this protein also localizes to the endosomes and lysosomes during these processes more studies will need to be done to understand the exact function of DdRab2 during these processes.

#### 3.2.2. Alterations in Development and Cell–Cell Cohesion

Several GTPases such as Rac1A [40], Rac1B [41], Rac1C [40], and RasGEFB [42] that have been implicated in vesicular trafficking have also shown to play a role in development. Rab2 has been identified as an essential protein for intracellular proliferation of *B. abortus* [43,44]. In mice, neurons treated with Rab2 show enhanced adhesion followed by a dramatic increase in neurite growth suggesting that Rab2 plays an important role in neuronal differentiation [45]. This led us to investigate the role of DdRab2 in development.

DdRab2(CA) and DdRab2(DN) expressing cells both displayed alterations in the developmental process and cell–cell adhesion. Both mutants were able to complete the developmental cycle however, they displayed an increase in the rate of development, forming fruiting bodies after only 16 h. The morphology of the fruiting bodies also differed from the AX4 cells. DdRab2(CA) cells had smaller fruiting bodies while the DN version had larger fruiting bodies compared to AX4 cells. Upon starvation, *Dictyostleium* cells will signal each other by releasing cAMP and aggregate together via chemotaxis to form a mound. An increased response to cAMP upon starvation would be a logical hypothesis to explain the increased rate of development in the DdRab2 mutants however, surprisingly, both DdRab2(CA), and DdRab2(DN) cells displayed a normal chemotactic response to cAMP (data not shown). Some *Dictyostelium* mutants are unable to aggregate normally by chemotaxis and do so by random motion and adhesion [42,54,55]. In most cases, although the resultant aggregates are smaller than normal, the time-scale of development is similar to that of the parental cells [42]. Another possible explanation for the increased rate of development observed in the CA and DN cells was due to altered cell–cell adhesion. We observed an initial increase in cell–cell adhesion of almost 40% after 20 min DdRab2(CA), and DdRab2(DN) cell lines. This increase in cell–cell adhesion leveled off after 40 min and was similar to AX4 cells. Interestingly, DdRab2(CA), and DdRab2(DN) cells showed a decrease after 20 min in EDTA-resistant cell–cell adhesion. Appearance of EDTA-resistant cell adhesion is due to the expression on the cell surface of the glycoprotein contact site A (csA). Failure to form EDTA-resistant adhesion is due to impaired expression on the cell surface of the csA glycoprotein [56]. It has been shown by Bozzaro 2006 [56], that a mutant defective in EDTA-stable adhesion, but displaying rather normal aggregation on agar, is, with very high probability, defective in functional csA. We believe that the defects in cell–cell adhesion seen in the CA and DN cell lines is due to improper signaling of downstream effectors. Perhaps one of the most surprising results of our developmental studies was the lack of differences seen in the CA and DN cell lines. In other GTPase family proteins, DN mutations have been shown to enhance binding to GTP exchange factors [41,57,58], a possible explanation for this is that DdRab2(CA), and DdRab2(DN) may act by primarily sequestering or mislocalizing DdRab2 associated proteins, in turn downregulating DdRab2 signaling pathways accounting for the similarity in CA and DN cells this is similar to results found by Palmieri, *et al.* 2000 [41] on GTPase Rac1. Endogenous DdRab2 is unable to access common effector molecules, therefore the inability to properly signal leads to the defects observed in the cells. We propose that for DdRab2 the ability to cycle between the active GTP-bound form and the inactive GDP-bound form is crucial for proper signaling of downstream effectors for cell–cell adhesion. The increased rate of development observed may be due to the cells being initially more cohesive allowing for quicker aggregation and mound formation and thus fruiting body formation. The slight phenotypic differences seen in the CA and DN cells during the different developmental phases may also be due to improper signaling to downstream effectors. DdRab2 over-expressing cells developed at the same rate and phenotypically similar to AX4 cells indicating that GTP/GDP hydrolysis of DdRab2 is required for proper *Dictyostelium* development.

#### 3.2.3. DdRab2 Plays a Role in Cell Death

Our findings that DdRab2 plays a role in development, specifically the very small fruiting bodies observed in the CA cell line, led us to investigate the role of DdRab2 in cell death. The fruiting body is composed of spore and stalk cells. The stalk cells are dead as the result of programmed cell death [59]. *Dictyostelium* is ideal for studying the mechanisms for autophagic cell death because the genome does not encode components of the apoptotic pathway [60]. In *Dictyostelium*, caspase activation is not required for cell death [28]. Apoptosis does not interfere with autophagic cell death in *Dictyostelium*; the organism instead displays developmental cell death [61]. Based on the phenotypes of the fruiting bodies displayed in the DdRab2 mutants, as well as the increased rate of development, we were curious if DdRab2 may contribute to developmental cell death.

Cell death was quantified in DdRab2 over-expressing, DdRab2(CA), and DdRab2(DN) cells using flow cytometry analysis as described previously. We found the DdRab2(CA) cells had a significantly reduced level of cell death of almost 20% less as compared to AX4 cells. DdRab2(DN) cells also showed a reduction in cell death of approximately 12%. We also measured the ATP levels of the cells as an indicator of metabolically active cells and found that DdRab2(CA) cells had an increased amount of metabolically active cells compared to AX4 cells correlating with the decrease in dead cells shown in Figure 6. Interestingly, DdRab2(DN) cells showed a decrease in metabolically active cells even though they showed a decrease in cell death. DdRab2(DN) cells also have a reduced rate of phagocytosis and pinocytosis so this may be the reason for the reduced metabolic activity. Over-expression of DdRab2 showed no significant difference in cell death compared to AX4 cells, however, it did have a slightly lower metabolic activity, also correlating to a decrease in phagocytosis and endocytosis. It appears that it is important for DdRab2 to maintain its GTP/GDP cycle for normal cell death as over-expression of DdRab2 appears not to have a significant effect on these cellular processes in *Dictyostelium*.

Mammalian Rab2 and the C.elegans UNC-108 (the mammalian Rab2 homolog) play important roles in the removal of apoptotic cells and promote phagosome maturation [53]. It is a possibility that a contributing factor to the reduction in apoptosis observed in DdRab2(CA) cells is due to these mutant cells having a better ability to remove apoptotic cells, thus less dead cells would be available for quantification. In a study by Lu, *et al.* 2008 [52] it was found that UNC-108/Rab2 in *C. elegans* had an increased number of cell corpses in *rab-2*(RNAi) animals. We do not however believe that this is the only factor, as DdRab2(DN) cells also show a decrease in cell death and they show a decrease in the rate of phagocytosis and pinocytosis. This indicates that DdRab2 may be indirectly involved in the process of cell death in *Dictyostelium.* When cells were assayed for their ability to re-grow after induced death, all cell lines showed an increase in re-growth. DdRab2 over-expressing, CA, and DN cell lines also showed an increase in growth rate in axenic media, directly correlating with their ability to re-grow after induced death. This data suggests that these cells may not have more cells surviving after induced death, but the cells that do survive are able to proliferate at a faster rate. More studies are needed to determine the exact role of DdRab2 in the process of cell death in *Dictyostelium*.

## 4. Conclusions

This is the first report describing a function for *Dictyostelium* Rab2 in vesicular trafficking, cell adhesion, development, and possibly cell death. These studies identify an envolvement for Rab protiens far more diverse than initially thought. While an increased rate of pinocytosis and phagocytosis was observed in the CA cell line, the DN version of the protien displayed the opposite effect. During the development and cell death studies, the results for the CA and DN cell lines appeared similar. We suggest that DdRab2 may be directly envolved in the processes of vesicluar trafficking, however it may be indirectly involved or unable to signal in the developmental processes and cell death. More studies will need to be done to understand the exact role for DdRab2.

## References

[1] Pfeffer S.R. (1994). Rab GTPases: Master regulators of membrane trafficking. Curr. Opin. Cell Biol..

[2] Schafer W.R., Rine J. (1992). Protein prenylation: Genes, enzymes, targets, and functions. Annu. Rev. Genet..

[3] Powell R.A., Temesvari L.A. (2004). Involvement of a Rab8-like protein of *Dictyostelium discoideum*, Sas1, in the formation of membrane extensions, secretion and adhesion during development. Microbiology.

[4] Grosshans B.L., Ortiz D., Novick P. (2006). Rabs and their effectors: Achieving specificity in membrane traffic. Proc. Natl. Acad. Sci. USA.

[5] Kypri E., Falkenstein K., de Lozanne A. (2013). Antagonistic control of lysosomal fusion by Rab14 and the Lyst-related protein LvsB. Traffic.

[6] Harris E., Cardelli J. (2002). RabD, a *Dictyostelium* Rab14-related GTPase, regulates phagocytosis and homotypic phagosome and lysosome fusion. J. Cell Sci..

[7] Harris E., Yoshida K., Cardelli J., Bush J. (2001). Rab11-like GTPase associates with and regulates the structure and function of the contractile vacuole system in *Dictyostelium*. J. Cell Sci..

[8] Chavrier P., Parton R.G., Hauri H.P., Simons K., Zerial M. (1990). Localization of low molecular weight GTP-binding proteins to exocytic and endocytic compartments. Cell.

[9] Lombardi D., Soldati T., Riederer M.A., Goda Y., Zerial M., Pfeffer S. (1993). Rab9 functions in transport between late endosomes and the trans Golgi network. EMBO J..

[10] Buczynski G., Bush J., Zhang L., Rodriguez-Paris J., Cardelli J. (1997). Evidence for a recycling role for Rab7 in regulating a late step in endocytosis and in retention of lysosomal enzymes in *Dictyostelium discoideum*. Mol. Biol. Cell.

[11] Nouffer C., Balch W.E. (1994). GTPases: Multifunctional molecular switches regulating vesicular traffic. Annu. Rev. Biochem..

[12] Green E.G., Ramm E., Riley N.M., Spiro D.J., Golenring J.R., Wesling-Rensick M. (1997). DdRab11 is associated with transferrin-containing recycling compartments in K562 cells. Biochem. Biophys. Res. Commun..

[13] Ren M., Xu G., Zeng J., DeLemos-Chiarandini C., Adesnik M., Sabatimi D.D. (1998). Hydrolysis of GTP on Rab11 is required for the direct delivery of transferrin from the pericentriolar recycling compartment to the cell surface but not from sorting endosomes. Proc. Natl. Acad. Sci. USA.

[14] Rupper A., Cardelli J. (2001). Regulation of phagocytosis and endo-phagosomal trafficking pathways in *Dictyostelium discoideum*. Biochem. Biophys. Acta.

[15] Bush J., Temesvari L., Rodriguez-Paris J., Buczynski G., Cardelli J. (1996). A role for a Rab4-like GTPase in endocytosis and in regulation of contractile vacuole structure and function in *Dictyostelium discoideum*. Mol. Biol. Cell.

[16] Peranen J., Auvinen P., Virta H., Wepf R., Simons K. (1996). Rab8 promotes polarized membrane transport through reorganization of actin and microtubules in fibroblasts. J. Cell Biol..

[17] Imamura H., Takaishi K., Nakano K., Kidama A., Oishi H., Shiozaki H., Monden M., Sasaki T., Takai Y. (1998). Rho and Rab small G proteins coordinately reorganize stress fibers and focal adhesions in MDCK cells. Mol. Biol. Cell.

[18] Hattula K., Furuhjelm J., Arffman A., Peranen J. (2002). A Rab8 specific GDP/GTP exchange factor is involved in actin remodeling and polarized membrane transport. Mol. Biol. Cell.

[19] Lau A.S., Mruk D.D. (2003). Rab8B GTPase and junction dynamics in the testis. Endocrinology.

[20] Zahraoui A., Joberty G., Arpin M., Fontaine J.J., Hellio R., Tavitian A., Louvard D. (1994). A small rab GTPase is distributed in cytoplasmic vesicles in non polarized cells but colocalizes with the tight junction marker ZO-1 in polarized epithelial cells. J. Cell Biol..

[21] Sheth B., Fontain J.J., Ponza E., McCallum A., Page A., Citi S., Louvard D., Zahraoui A., Fleming T.P. (2000). Differentiation of the epithelial apical junctional complex during mouse preimplantation development: A role for Rab13 in the early maturation of the tight junction. Mech. Dev..

[22] Marzesco A.M., Dunia I., Pandjaitan R., Recouvreur M., Dauzonne D., Benedetti E.L., Louvard D., Zahraoui A. (2002). The small GTPase Rab13 regulates assembly of functional tight junctions in epithelial cells. Mol. Biol. Cell.

[23] Chen Y.T., Holcomb C., Moore H.P. (1993). Expression and localization of two low molecular weight GTP-binding proteins, Rab8 and Rab10, by epitope tag. Proc. Natl. Acad. Sci. USA.

[24] Vadlamudi R.K., Wang R.A., Talukder A.H., Adam L., Johnson R., Kumar R. (2000). Evidence of Rab3A expression, regulation of vesicle trafficking, and cellular secretion in response to heregulin in mammary epithelial cells. Mol. Cell Biol..

[25] Loomis W.F. (1975). Dictyostelium Discoideum: A Developmental System.

[26] Chisholm R., Firtel R.A. (2004). Insights into morphogenesis from a simple developmental system. Nat. Rev. Mol. Cell Biol..

[27] Raper K. (1935). Dictyostelium discoideum, A new species of slime mold from decaying forest leaves. J. Agr. Res..

[28] Ollie R.A., Durrieu F., Cornillon S., Loughran G., Gross J., Earnshaw W.C., Golstein P. (1998). Apparent caspase independence of programmed cell death in *Dictyostelium*. Curr. Biol..

[29] Cornillon S., Foa C., Davoust J., Buonavista N., Gross J.D., Golstein P. (1994). Programmed cell death in *Dictyostleium*. J. Cell Sci..

[30] Fey P., Kowal A.S., Gaudet P., Pilcher K.E., Chisholm R.L. (2007). Protocols for growth and development of Dictyostelium discoideum. Nat. Protoc..

[31] Bush J., Nolta K., Rodriguez-Paris J., Kaufmann N., O’Halloran T., Ruscetti T., Temesvari L., Steck T., Cardelli J. (1994). A Rab4-like GTPase colocalizes with V-H^+^-ATPases in extensive reticular elements of the contractile vacuoles and lysosomes in *Dictyostelium discoidem*. J. Cell Sci..

[32] Rivero F., Maniak M. (2006). Quantitative and microscopic methods for studying the endocytic pathway. Methods Mol. Biol..

[33] Rodriguez-Paris J.M., Nolta K.V., Steck T.L. (1993). Characterization of lysosomes isolated from *Dictyostelium discoideum* by magnetic fractionation. J. Biol. Chem..

[34] Klein G., Satre M. (1986). Kinetics of fluid-phase pinocytosis in *Dictyostelium discoideum* amoebae. Biochem. Biophys. Res. Commun..

[35] Wong E., Yang C., Wang J., Fuller D., Loomis W.F., Siu C.H. (2002). Disruption of the gene encoding the cell adhesion molecule DdCAD-1 leads to aberrant cell sorting and cell-type proportioning during *Dictyostelium* development. Development.

[36] Secko D.M., Siu C.H., Spiegelman G.B., Weeks G. (2006). An activated Ras protein alters cell adhesion by dephosphorylating *Dictyostelium* DdCAD-1. Microbiology.

[37] Desbarats L., Brar S.K., Siu C.H. (1994). Involvement of cell-cell adhesion in the expression of the cell cohesion moelcule GP80 in *Dictyostelium discoideum*. J. Cell Sci..

[38] Hannah R., Micheal B., Moravec R. (2001). Cell-Titer-GLO luminescent cell viability: A sensitive and rapid method for determining cell viability. Promega Cell Notes.

[39] Lam D., Levraud J.P., Luciani M.F., Golstein P. (2007). Autophagic or necrotic cell death in the absence of caspase and Bcl-2 family members. Biochem. Biophys. Res. Commun..

[40] Dumontier M., Höcht P., Mintert U., Faix J. (2000). Rac1 GTPases control filopodia formation, cell motility, endocytosis, cytokinesis and development in *Dictyostelium*. J. Cell Sci..

[41] Palmieri S.J., Nebl T., Pope R.K., Seastone D.J., Lee E., Hinchcliffe E.H., Sluder G., Knecht D., Cardelli J., Luna E.J. (2000). Mutant Rac1B expression in *Dictyostelium*: Effects on morphology, growth, endocytosis, development, and the actin cytoskeleton. Cell Motil. Cytoskeleton.

[42] Wilkins A., Chubb J.R., Insall R.H. (2000). A novel *Dictyostelium* RasGEF is required for normal endocytosis, cell motility and multicellular development. Curr. Biol..

[43] Fugier E., Salcedo S.P., de Chastellier C., Pophillat M., Muller A., Arce-Gorvel V., Fourquet P., Gorvel J.P. (2009). The glyceraldehyde-3-phosphate dehydrogenase and the small GTPase Rab 2 are crucial for Brucella replication. PLoS Pathog..

[44] deBarsy M., Jamet A., Filopon D., Nicolas C., Laloux G., Rual J.F., Muller A., Twizere J.C., Nkengfac B., Vandenhaute J. (2011). Identification of a Brucellaspp. secreted effector specifically interacting with human small GTPase Rab2. Cell Microbiol..

[45] Ayala J., Touchot N., Zahraouit A., Tavitian A., Prochiantz A. (1990). The product of Rab2, a small GTP binding protein, increases neuronal adhesion, and neurite growth *in vitro*. Neuron.

[46] Norian L., Dragoi I.A., O’Halloran T. (1999). Molecular characterization of RabE, a developmentally regulated *Dictyostelium* homolog of mammalian Rab GTPases. DNA Cell Biol..

[47] Kawauchi T. (2011). Regulation of cell adhesion and migration in cortical neurons: Not only Rho but also Rab family small GTPases. Small GTPases.

[48] Kay R.R., Berks M., Trayner D. (1989). Morphongen hunting in *Dictyostelium discoideum*. Development.

[49] Zerial M., McBride H. (2001). Rab proteins as membrane organizers. Nat. Rev. Mol. Cell Biol..

[50] Chun D.K., McEwen J.M., Burbea M., Kaplan J.M. (2008). UNC-108/Rab2 regulates postendocytic trafficking in *Caenorhabditis elegans*. Mol. Biol. Cell.

[51] Stenmark H., Olkkonen V.M. (2001). The Rab GTPase family. Genome Biol..

[52] Lu Q., Zhang Y., Hu T., Guo P., Li W., Wang X. (2008). *C. elegans* Rab GTPase 2 is required for the degradation of apoptotic cells. Devlopment.

[53] Mangahas P.M., Yu X., Miller K.G., Zhou Z. (2008). The small GTPase Rab2 functions in the removal of apoptotic cells in *Caenorhabditis elegans*. J. Cell Biol..

[54] Ma H., Gamper M., Parent C., Firtel R.A. (1997). The *Dictyostelium* MAP kinase kinase DdMEK1 regulates chemotaxis and is essential for chemoattractant-mediated activation of guanylyl cyclase. EMBO J..

[55] Vithalani K.K., Parent C.A., Thorn E.M., Penn M., Larochelle D.A., Devreotes P.N., de Lozanne A. (1998). Identification of darlin, a *Dictyostelium* protein with armadillo-like repeats that binds to small GTPases and is important for the proper aggregation of developing cells. Mol. Biol. Cell.

[56] Bozzaro S., Ludwig E., Rivero F. (2006). Assaying cell-cell adhesion. Dictyostelium Discoideum Protocols.

[57] Fransworth C.L., Feig L.A. (1991). Dominant inhibitory mutations in the Mg^2+^-binding site of RasH prevent its activation by GTP. Mol. Cell Biol..

[58] Burstein E.S., Brondyk W.H., Macara I.G. (1992). Amino acid residues in the Ras-like GTPase Rab3A that specify sensitivity to factors that regulate the GTP/GDP cycling of Rab3A. J. Biol. Chem..

[59] Kosta A., Laporte A., Lam D., Tresse E., Luciani M.F., Golstein P., Eichinger L., Rivero F. (2006). How to asses and study cell death in *Dictyostelium discoideum*. Dictyostelium Discoideum Protocols.

[60] Kourtis N., Tevernarakis N. (2009). Autophagy and cell death in model organisms. Cell Death Differ..

[61] Whittingham W.F., Raper K.B. (1960). Non-viability of stalk cells in dictyostelium. Proc. Natl. Acad. Sci. USA.

